# Reduced Face Preference in Infancy: A Developmental Precursor to Callous-Unemotional Traits?

**DOI:** 10.1016/j.biopsych.2014.09.022

**Published:** 2015-07-15

**Authors:** Rachael Bedford, Andrew Pickles, Helen Sharp, Nicola Wright, Jonathan Hill

**Affiliations:** a Biostatistics Department, Institute of Psychiatry, King’s College London, London; b Institute of Psychology, Health and Society, University of Liverpool, Liverpool; c Centre for Developmental Science and Disorders, University of Manchester, Manchester, United Kingdom.

**Keywords:** Callous-unemotional traits, Face preference, Maternal sensitivity, Infant development, Precursor, Psychopathy

## Abstract

**Background:**

Children with callous-unemotional (CU) traits, a proposed precursor to adult psychopathy, are characterized by impaired emotion recognition, reduced responsiveness to others’ distress, and a lack of guilt or empathy. Reduced attention to faces, and more specifically to the eye region, has been proposed to underlie these difficulties, although this has never been tested longitudinally from infancy. Attention to faces occurs within the context of dyadic caregiver interactions, and early environment including parenting characteristics has been associated with CU traits. The present study tested whether infants’ preferential tracking of a face with direct gaze and levels of maternal sensitivity predict later CU traits.

**Methods:**

Data were analyzed from a stratified random sample of 213 participants drawn from a population-based sample of 1233 first-time mothers. Infants’ preferential face tracking at 5 weeks and maternal sensitivity at 29 weeks were entered into a weighted linear regression as predictors of CU traits at 2.5 years.

**Results:**

Controlling for a range of confounders (e.g., deprivation), lower preferential face tracking predicted higher CU traits (*p* = .001). Higher maternal sensitivity predicted lower CU traits in girls (*p* = .009), but not boys. No significant interaction between face tracking and maternal sensitivity was found.

**Conclusions:**

This is the first study to show that attention to social features during infancy as well as early sensitive parenting predict the subsequent development of CU traits. Identifying such early atypicalities offers the potential for developing parent-mediated interventions in children at risk for developing CU traits.

Among antisocial adults, psychopathy is associated with more severe and enduring violence and offending (1, 2). The proposed childhood equivalent, callous-unemotional (CU) traits, evidenced by a lack of responsiveness to, or concern for, others’ distress, is also associated with severity and persistence of offending and aggression as well as increased burden on families (3, 4, 5). Despite the clear need to understand the developmental origins of CU traits, few studies have examined infant predictors.

In children with CU traits, core impairments such as responsiveness to others’ distress cues have been associated with a lack of attention to the eye region. Eye contact occurs during interaction with a parent or caregiver and forms a critical component of an infant’s early social communication, influencing the development of the social brain (6). The early environment also plays an important role with factors such as positive parenting associated with lower CU traits, offering a target for potential interventions. The present study examines the role of infants’ preferential attention to a face with direct gaze and maternal sensitivity as predictors of subsequent CU traits.

### Attention to the Face

In typical development, early social interaction (e.g., attention to faces, reciprocal smiling) facilitates bonding with (and learning from) the caregiver during the protracted period of postnatal development (7, 8). In typical development, sensitivity to another person’s face and eye gaze appears to be present from immediately after birth (8, 9). In the first few months of life, faces are processed by a subcortical route, via the superior colliculus, pulvinar, and amygdala (10). Infant face preference is thought to be driven by the configuration of the eyes within the face, which maximally activates this low spatial frequency subcortical pathway (11). More recently, this route has been proposed to modulate activation and influence the development of the social brain network [i.e., fusiform gyrus, prefrontal cortex, and superior temporal sulcus regions (6)] specialized in adults for the processing of social stimuli. It is plausible that atypical face preferences early in infancy contribute to later impairments in socioemotional behavior (12).

A decreased recognition of, and amygdala activation in response to, fearful faces, which may underpin failures to regulate aggression in the face of others’ distress, is associated with CU traits (13). There is also evidence that elevated CU traits and reduced fear recognition are associated with reduced attention to the eyes (14, 15, 16, 17). Could early difficulties in orienting toward a person’s face underlie the social and emotional impairments characteristic of children with CU traits? Dadds *et al.* (16) hypothesized that a failure to attend to emotionally salient stimuli such as another person’s eyes might be present very early in the development of CU traits, with direct effects on responsiveness to others’ distress and indirect effects through a reduced ability to benefit from parental sensitivity. However, to date, no studies have examined whether reduced face preference in infancy could represent a developmental precursor to CU traits.

### Role of Parenting in CU Traits

A child’s early environment is known to play a role in the development of CU traits (18). Research into the effects of early parent characteristics on subsequent CU traits has yielded mixed results; several studies failed to show associations for measures such as parental involvement, monitoring/supervision, discipline, and poor parental practices (19, 20, 21). However, associations have been found between early positive parenting characteristics (i.e., parental warmth) and decreased CU traits (22, 23, 24) [Waller *et al.* (25)]. Low maternal warmth and affection predict higher adolescent CU traits even after controlling for risk factors such as childhood abuse and neglect (26). Interactions with parents over the first months of life provide numerous opportunities to attune with others’ emotions and intentions, provided that parents are responsive to infant cues (27, 28). Sensitive parenting may promote the development of empathy and social understanding and reduce risk of CU traits.

### Dyadic Interaction

There may be limits to the effect of parental sensitivity in the presence of reduced looking toward the caregiver’s face. Dadds *et al.* (16) specifically hypothesized that an early lack of attention to the eyes of an attachment figure reduces the beneficial effects of sensitive parenting and, over the course of development, results in the social and empathy deficits characteristic of children with CU traits. In line with this hypothesis, they found reduced eye contact in boys with high CU traits (age range, 5–16 years; mean, 8.9 years) in interactions with both of their parents, despite the fact that the mothers of the children with high CU traits did not themselves show impairments, a finding replicated in a later study by Dadds *et al.* (17).

### Gender Differences in CU Traits

Most studies of CU traits have focused predominately on male participants, likely because of increased prevalence of CU traits in boys (29). However, several studies have found gender differences relating to the relative contribution of genetic and shared environmental influences suggesting that CU traits in boys are more highly heritable than CU traits in girls (30, 31). Gender differences have been found for parenting, with positive parenting measures negatively associated with CU traits only in girls (22, 23). Exploring the role of gender in the developmental pathway leading to CU traits is an important next step.

This is one of the first studies to examine prospectively early developmental processes in CU traits. Based on available evidence from cross-sectional studies in children, we hypothesized that reduced preferential tracking of a face with direct gaze in infancy represents an early vulnerability for CU traits. In addition, early parental sensitivity may lead to reductions in CU traits by increasing opportunities to respond to others’ emotions and intentions. However, the beneficial effect of parental sensitivity on the development of CU traits may be reduced by lower eye contact and may be less evident in boys than in girls. We tested the following specific predictions: 1) reduced preferential tracking of the human face at 5 weeks will be associated with higher CU traits at 2.5 years, 2) elevated maternal sensitivity assessed at 29 weeks will predict lower CU traits, 3) there will be an interaction between maternal sensitivity and face tracking in the prediction of CU traits, and 4) there will be an interaction between maternal sensitivity and sex of infant in the prediction of CU traits.

## Methods and Materials

### Participants

Mothers and children participated in the Wirral Child Health and Development Study, a consecutive cohort of 1233 women (mean age, 26.8 years; SD, 5.8 years) recruited at 20 weeks of pregnancy with their first child [Sharp *et al.* (32)]. An intensive subsample of 316 women, stratified by partner psychological abuse, was identified at 32 weeks’ gestation. The extensive general population sample consisted of all participants. For the current study, data were analyzed from 213 (105 male; 108 female) participants in the intensive sample who had data at “32 weeks prenatal” (mean weeks of pregnancy, 32.1; SD, 2.1) and at postnatal assessment points, as follows: “5 weeks” (mean, 5.2; SD, 1.1 weeks), “29 weeks” (mean, 29.1; SD, 3.1 weeks), and “2.5 years” (mean, 31.4; SD, 2.5 months) (Figure S1 in Supplement 1).

### Procedure and Measures

#### Neonatal Behavioral Assessment Scale

The Neonatal Behavioral Assessment Scale (NBAS), a standardized measure designed to assess orienting, motor, and emotion regulatory processes (33, 34), was administered to the intensive sample at 5 weeks after birth in the laboratory. It was conducted by a trained administrator who carried out, in a prescribed sequence, a range of maneuvers designed to elicit the infant’s optimal orienting and motor performance and emotional responses to mildly aversive procedures. There are six orienting procedures during which the infant is positioned on the administrator’s lap at 45 degrees, with the infant’s head supported by the administrator. However, because the focus of this study was face preference, we used only two subscales: 1) orientation to the human face, assessed as the extent to which the infant moves eyes and head to track the administrator’s face over a 180-degree horizontal arc and 30 degrees vertically and 2) orientation to an inanimate visual stimulus, a red ball (while the administrator keeps his or her face out of the infant’s line of vision). To separate out the effects of infant alertness and general interest and maturity from preferential interest in faces, we transformed the two standardized orientation scores into a face/ball mean, which indicated general tracking ability, and face/ball difference, indicating specific preference for the face.

Ratings were made by the administrator from memory immediately after the assessment. To assess interrater reliability, the assessments were video recorded using four cameras placed to obtain a comprehensive picture of infant responses. Three assessors were trained by Dr. Joanna Hawthorne, director of the Brazelton Centre in the United Kingdom. Pair-wise agreement (intraclass correlation coefficient) between independent ratings made from memory and video recordings on 220 (out of the full intensive sample seen at 5 weeks, *N* = 260) were .77 for face tracking and .75 for tracking of the red ball.

#### Maternal Sensitivity

Maternal sensitivity was assessed when infants were 29 weeks old with a 15-min standard laboratory-based procedure (35). Mothers were asked to play with their infants as they would at home for 7 minutes with toys supplied by the mother and for 8 minutes with a standard set of toys provided by the experimenter. Maternal sensitivity was rated from video recordings on a global 5-point scale, ranging from 1 (not at all characteristic) to 5 (highly characteristic) reflecting mothers’ appropriate, supportive, warm responding to infant communications, playful bids or distress. Training on the sensitivity measure was provided by an investigator from the National Institute of Child Health and Human Development Network. Three raters, blind to the other measures, coded sensitivity from video recordings. Each rater achieved good interrater reliability for maternal sensitivity on a subset of 30 assessments (intraclass correlation coefficients .85–.91).

#### CU Traits

CU traits were assessed via parental questionnaire when the children were 2.5 years old. The Antisocial Process Screening Device (APSD) CU traits subscale is a widely used measure of CU traits, but this measure has low internal reliability (5). To create a more reliable measure of CU traits, Dadds *et al.* (5) ran a factor analysis on the APSD CU traits subscale and additional items from the Strengths and Difficulties Questionnaire. Following this approach, in the current study we combined items from the APSD with items from the developmentally appropriate Child Behavior Checklist (CBCL) (36, 37) and an additional item from the Brief Infant Toddler Social Emotional Assessment chosen for its similarity to the prosocial Strengths and Difficulties Questionnaire items (35). Item responses for each questionnaire were 0, 1, 2, not true, sometimes true, or very true.

To derive a CU traits factor (Table 1), exploratory factor analysis for ordinal data (using a weighted least squares means adjusted estimator and Promax rotation) was undertaken in Mplus (38). The APSD item “He/She … Does not show feelings or emotions” was removed because of problems with empty cells in the cross-tabulation with CBCL items “Shows little affection toward people” and “Seems unresponsive to affection.” Examination of the scree plot offered support for a one-factor solution (eigenvalues for first factor = 4.7, second = 1.6, third = 1.1). The only item loading <.35 was CBCL “Shows too little fear of getting hurt.” All other items were included in a confirmatory factor analysis (items are indicated in Table 1) using a weighted least squares means and variances adjusted estimator and factor scores derived for each participant. The model fit statistics suggested a reasonable fit to the data (root mean square error of approximation = .09, comparative fit index = .87). The internal reliability of this combination of items yielded a much higher Cronbach α value (.69) than the APSD items alone (.53).

**Table 1:** Source Questionnaire, Item Numbers, and Question Wording for Items Included in CU Trait Factor Analysis

APSD	CBCL	BITSEA
1. He/She … Is unconcerned about the feelings of others^a^	14. Cruel to animals^a^	22. Tries to help when someone is hurt (for example, gives a toy)^a^
2. He/She … Seems motivated to do his/her best in structured activities^a^		
3. He/She … Is good at keeping promises^a^	58. Punishment doesn’t change his/her behavior^a^	
4. He/She … Feels bad or guilty when he/she does something wrong^a^	67. Seems unresponsive to affection^a^	
5. He/She … Keeps the same friends^a^	69. Selfish or won’t share^a^	
6. He/She … Does not show feelings or emotions	70. Shows little affection toward people^a^	
	72. Shows too little fear of getting hurt	

All potential items relating to CU traits from the CBCL, APSD, and BITSEA.

APSD, Antisocial Process Screening Device; BITSEA, Brief Infant Toddler Social and Emotional Assessment; CBCL, Child Behavior Checklist; CU, callous-unemotional.

*a* Item was retained in the final confirmatory factor analysis.

#### Confounders

Demographic and biological risks known to be associated with NBAS performance and with child mental health disorders (39) were included as potential confounders; these included age at NBAS, gestational age (from birth records), smoking during pregnancy (derived from information obtained at 20 weeks of gestation in the extensive sample and again at 32 weeks in the intensive sample only), maternal antisocial traits (Maternal Antisocial Personality Disorder total symptom scores from the Structured Clinical Interview for DSM-IV Personality Disorders) and socioeconomic status [the English Index of Multiple Deprivation (40)]. Participants were ranked according to their area postal code and assigned to a quintile based on the United Kingdom distribution of deprivation).

### Statistical Analysis

Factor scores from the confirmatory factor analysis model (see earlier section on CU traits) were extracted and linear regression models fitted to assess their association with the predictor variables. The two-phase stratified sample design allowed estimates to be reported for the general population from the stratified subsample (intensive sample) by using inverse sampling probability weights. Weights took account not only of the original stratification but also of the sample attrition up to the assessment at age 2.5 years including mothers’ age and years of education, maternal smoking and depression score in pregnancy, and a score of the number of items left incomplete at the initial assessment. Variation in the weights associated with the covariates of each model was removed to improve efficiency.

Analyses were done in Stata 12 (41). Statistics for weighted means, correlations (using aweights) and regression estimates (using pweights) are based on survey adjusted Wald tests (*t* tests if single degrees of freedom or *F* tests if multiple degrees of freedom) using the robust “sandwich” estimator of the parameter covariance matrix (42).

## Results

### Sample Description

Table 2 presents descriptive statistics for the sample. Noteworthy are the higher CU scores for boys compared with girls at age 2.5 years and the modest tendency to orient to the face more than the ball at 5 weeks.

**Table 2:** Sample Weighted Descriptive Statistics for the Population of Infants

	Male	Female
	Mean, SD (*n*)	Mean, SD (*n*)
CU Trait Factor Score	.13, .48 (112)	−.77, .43 (112)
Face Tracking	7.3, 1.37 (112)	7.46, .97 (112)
Ball Tracking	6.62, 1.81 (112)	6.68, 2.07 (112)
Face/Ball Difference	.50, .97 (112)	.45, 1.03 (112)
Maternal Sensitivity	3.61, .98 (105)	3.60, .97 (108)
Smoking in Pregnancy	.58, .82 (112)	.53, .75 (112)
Deprivation Quintile	2.36, 1.24 (112)	2.16, 1.30 (112)
Mother’s APSD	.02, .98 (112)	−.18, .98 (112)
Gestational Age at Birth	40.07, 1.03 (112)	40.28, 1.52 (112)

APSD, Antisocial Process Screening Device; CU, callous-unemotional.

### Preferential Face Tracking and Maternal Sensitivity

Initially, we ran a simple model looking at the prediction of CU traits at 2.5 years by 5-week face and ball tracking. As expected, increased tracking of the face was associated with lower CU traits (coefficient [coef] = −.10, SE = .035, *p* = .004). Conversely, tracking of the ball was significantly positively associated with CU traits (coeff = .10, SE = .035, *p* = .004). All subsequent analyses look at the face/ball mean and difference scores to assess preferential face tracking (rather than general ability to orient to a stimulus).

We examined how, in a linear regression model, CU traits were predicted by the face/ball mean and difference scores, maternal sensitivity, sex, and age at NBAS (because infants varied in testing age; mean, 5.2 weeks, SD, 1.1 weeks). The face/ball mean score was not a significant predictor (coeff = .027, SE = .037, *p* = .46), but difference score was associated with later CU traits (coeff = −.10, SE = .031, *p* = .001) with increased face tracking predicting lower CU traits. Increased maternal sensitivity also predicted lower CU trait levels (coeff = −.082, SE = .037, *p* = .03). The control variables, sex (coeff = .21, SE = .073, *p* = .004) and age at NBAS (coeff = .011, SE = .005, *p* = .03), were significant with higher CU traits in boys compared with girls and in infants assessed later on the NBAS.

**Figure 1:**
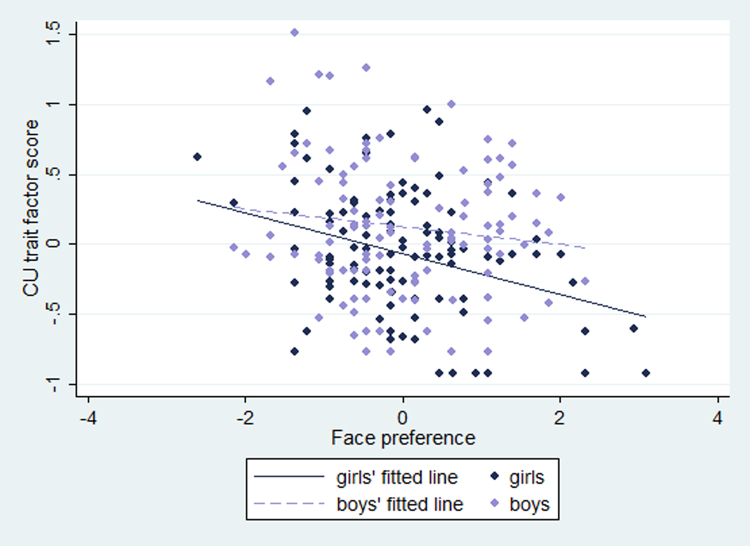
Relationship between 5-week face/ball difference score and 2.5-year callous-unemotional traits. CU, callous-unemotional.

When we ran the regression model for the sexes pooled together but including interactions with sex, neither face/ball difference*sex (coeff = .012, SE = .033, *p* = .72) nor maternal sensitivity*sex (coeff = .028, SE = .036, *p* = .45) interactions were significant. Results for separate sexes should be interpreted with caution. However, when we looked at the sexes separately, we found for girls the relationship of CU traits with face/ball difference score (coeff = −.097, SE = .035, *p* = .007) and with maternal sensitivity (coeff = −.12, SE = .038, *p* = .002) remained strong, whereas for boys neither relationship reached significance: face/ball difference (coeff = −.058, SE = .055, *p* = .29) and maternal sensitivity (coeff = −.073, SE = .059, *p* = .22) (Figure 1). We then ran the model again to include an interaction term between face/ball difference score and maternal sensitivity, which was not significant for boys (coeff = −.066, SE = .048, *p* = .17) or girls (coeff = .031, SE = .034, *p* = .36).

### Effect of Confounders

Finally, we ran a combined linear regression model including the possible confounding effects of maternal smoking, antisocial personality trait score, socioeconomic status, and gestational age at birth. Results remained substantively similar: In the overall sample, all confounders other than smoking (coeff = .089, SE = .043, *p* = .04) were nonsignificant (*p* > .05). The face/ball difference score remained significant (coeff = −.11, SE = .032, *p* = .001), although the effect of maternal sensitivity became nonsignificant (coeff = −.057, SE = .039, *p* = .15). The confounder analysis was then run for boys and girls separately. For girls, face/ball difference (coeff = −.085, SE = .037, *p* = .02) and maternal sensitivity (coeff = −.12, SE = .043, *p* = .009) were significant, but none of the predictors remained significant for the boys (Table 3).

**Table 3:** Weighted Regression Prediction of CU Factor Scores Including Confounders

	Overall (*N* = 213)	Male (*n* = 105)	Female (*n* = 108)
	Coeff, (Robust SE)	Coeff, (Robust SE)	Coeff, (Robust SE)
Face/Ball Difference	−.107^a^ (.032)	−.066 (.055)	−.085^a^ (.037)
Maternal Sensitivity	−.057 (.039)	−.005 (.058)	−.116^a^ (.043)
Face/Ball Mean	.008 (.036)	.008 (.045)	.084 (.057)
Age at NBAS	.012^a^ (.004)	.017^a^ (.006)	<.001 (.004)
Sex	.198^a^ (.071)	—	—
Smoking in Pregnancy	.089^a^ (.043)	.049 (.058)	.098 (.06)
Deprivation Quintile	−.014 (.030)	−.079 (.048)	.043 (.026)
Mother’s APSD	.005 (.042)	−.008 (.06)	.032 (.043)
Gestational Age at Birth	.041 (.024)	.018 (.041)	.029 (.018)

APSD, Antisocial Process Screening Device; Coeff, coefficient; CU, callous-unemotional; NBAS, Neonatal Behavioral Assessment Scale.

*a* Significant difference.

To check whether results were specific to our measure of CU traits, we also ran the analysis using the APSD questionnaire as the outcome (rather than our factor score). Results remained similar with significant overall effects for face/ball difference score (coeff = −.29, SE = .13, *p* = .025) and maternal sensitivity (coeff = −.31, SE = .14, *p* = .031) on later APSD scores.

## Discussion

Although reduced attention to the caregiver’s face and the eye region in particular has been proposed to underlie the social and emotional impairments that characterize CU traits (16), this is the first study to examine the developmental origins of this behavior during infancy. Our aim was to test whether infants’ face preference and maternal sensitivity were related to subsequent CU traits and whether these relationships held after accounting for known risk factors (e.g., deprivation). In line with our hypotheses, we found that lower preferential tracking of a face with direct gaze versus a red ball was predictive of later increased CU traits, even after controlling for confounders. Although no significant interactions with gender were found and any sex difference results should be interpreted with caution, breaking the analysis down by gender showed this relationship may be stronger in girls. Maternal sensitivity also predicted CU traits, although the relationship remained significant only for girls when confounders were included in the model. However, we did not find support for the hypothesis that the beneficial effect of maternal sensitivity on CU traits is reduced in the presence of reduced face preference.

Our findings provide a critical extension to previous studies by demonstrating that reduced preference for a face with direct gaze versus a nonsocial object at just 5 weeks of age is associated with higher CU trait scores at 2.5 years. The subcortical route mediating preferential face tracking in typical development (8, 43) interacts with the development of specialized “social brain” regions (6) offering a potential mechanism by which a relatively basic perceptual bias can influence subsequent social-cognitive processing (12, 44).

What might such later deficits entail? It is well established in the literature that psychopathic individuals and children with high CU traits have impairments in fear recognition owing to a lack of attention to the relevant social features, particularly the eyes. Processing information from the eye region is particularly important for recognizing fearful facial expressions, and according to Blair (45), this attention to distress cues is a prerequisite for the development of morality and feelings of empathy and guilt. He proposed that an early failure in the “violence inhibition mechanism” and concomitant lack of attention to these cues results in a failure to inhibit aggressive responses in the context of another’s distress.

Although reduced attention to salient social features in infancy could potentially result in the behaviors associated with CU traits, the etiology of this reduced face preference is unknown. Although characterizing the neural underpinning is beyond the scope of the present study, it is reasonable to hypothesize a role for the amygdala. The amygdala, superior colliculus, and pulvinar comprise the subcortical visual pathway responsible for face preference in typical newborn infants. Limbic activation, particularly in the amygdala, is thought to be important for recognizing fearful facial expressions (46). The amygdala is strongly activated by the large white sclera of the eyes, a characteristic feature of fearful faces (47). Both amygdala-lesioned patients and children with high CU traits show fear recognition deficits that normalize on explicit instruction to attend to the eye region (48). Damage to the amygdala early in development has cascading effects across development resulting in later impairments in empathy (49).

Our finding that maternal sensitivity is negatively associated with CU traits in girls suggests that environmental factors such as parenting can influence the developmental pathway leading to CU traits. Results are consistent with previous studies in which positive parenting and parental warmth predicted decreased CU trait levels (23, 24). Hawes *et al.* (23) found a moderating effect of gender on the relationship between parenting and CU traits, with positive parenting negatively associated with CU traits, particularly in girls. Similarly, parental warmth has been associated with a reduction in CU trait outcomes for girls, but not for boys (22), although Pasalich *et al.* (50) showed that parental warmth was negatively associated with conduct problems in boys who had higher CU trait levels. Although we know from twin studies that CU traits are highly heritable, the magnitude of shared environmental influences on CU traits differs between boys and girls (30, 31) potentially suggesting that in girls early environmental factors play a greater role in the development of CU traits.

It is important to consider the possibility that our parental measures may reflect markers of genetic risk. For example, high levels of maternal sensitivity may be present only in parents who have low CU traits themselves, although by controlling for maternal ASPD scores (from Structured Clinical Interview for DSM-IV Personality Disorders), we have attempted to minimize this possibility. In addition, the use of an observational measure of maternal sensitivity in the laboratory may not generalize to day-to-day contexts. Another limitation concerns the use of the NBAS to assess face tracking. Although this measure benefits from several advantages, such as its broad clinical usage and high ecological validity compared with more traditional experimental measures, it cannot be used to tease apart more fine-grained aspects of infants’ looking behavior (e.g., fixations to the eyes vs. mouth). In the future, studies using real-world eye tracking will be important for establishing exactly where infants fixate when tracking a face. In addition, we do not know whether the current findings relate to social versus nonsocial stimuli in general or more specifically to face preference. To address this, manipulations of computer-based experimental stimuli (e.g., face with direct gaze vs. averted gaze or eyes closed) will be required. Finally, although several studies have confirmed the validity of measuring CU traits in preschool-age children (36, 51), further work is required to determine the longitudinal stability of CU traits at these early ages.

Another important area for future research will be to extend the current approach by assessing the overlap with co-occurring disorders (e.g., attention-deficit/hyperactivity disorder) and disorders with shared symptoms (e.g., autism spectrum disorder, which is characterized by social interaction difficulties including atypical attention to faces). Infant studies tend to find typical or even increased looking time to faces in infants who later develop autism (52, 53, 54, 55). Characterizing the common and distinct pathways across traits of different disorders will be important to determine the specificity of developmental relationships to CU traits.

In conclusion, this is the first study to show that decreased preferential tracking of the human face soon after birth is associated with later CU traits. This effect was slightly stronger in girls, whereas the beneficial effect of maternal sensitivity was significant only in girls, consistent with the emerging idea that developmental mechanisms in CU traits may differ between boys and girls (56, 57). The results support the hypothesis that reduced attention to social features in infancy can have downstream consequences for the development of socioaffective behaviors. Given the high economic and societal cost of CU traits (increased burden on families, higher rates of marital discord, criminality, and antisocial behavior), understanding the role of parental sensitivity and infant social attention provides a critical first step toward developing early parent-based interventions.
