# Supplementary material for: Reduced Face Preference in Infancy: A Developmental Precursor to Callous-Unemotional Traits?
Source: Biol Psychiatry. 2015 Jul 15;78(2):144–50. doi: 10.1016/j.biopsych.2014.09.022 (PMC4510143; doi:10.1016/j.biopsych.2014.09.022)
Supplement: Supplementary file 1 — Supplementary Material [file mmc1.pdf]

Reduced Face Preference in Infancy:
A Developmental Precursor to Callous-Unemotional Traits?

Supplementary Information

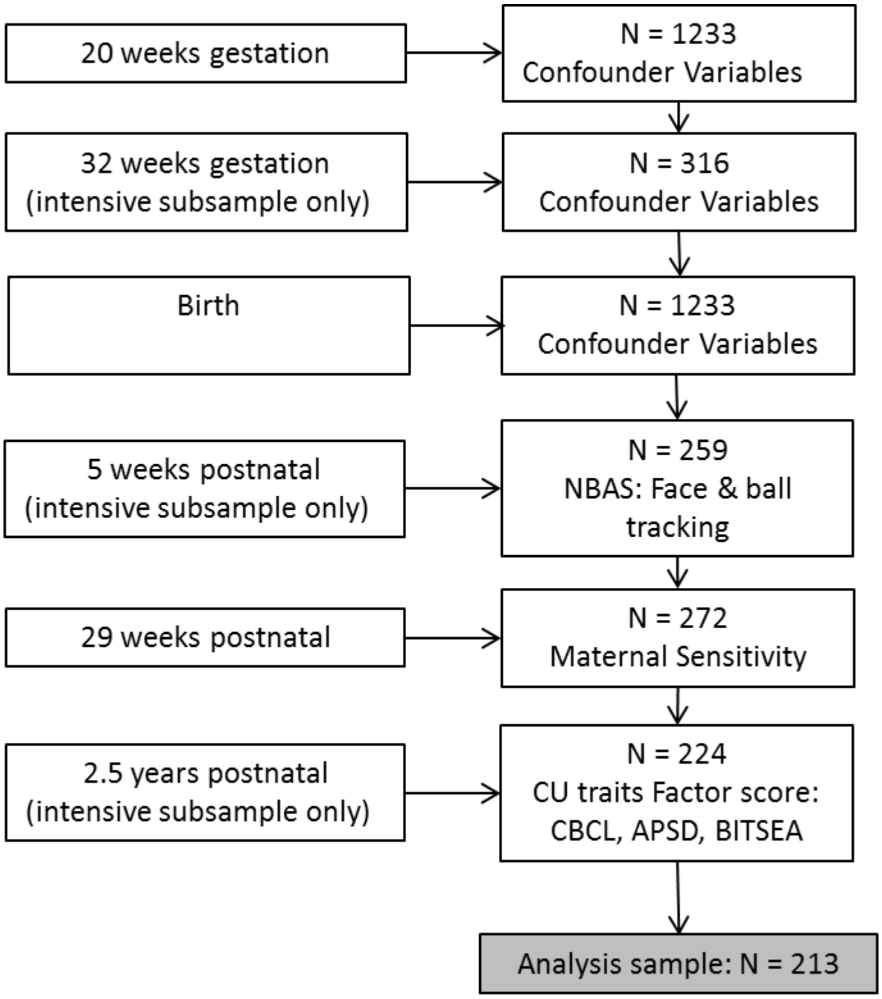

Figure S1. Flow Diagram. Participant numbers at each phase, and those entered into the final analysis.
